# Genome-Wide Identification, Comprehensive Gene Feature, Evolution, and Expression Analysis of Plant Metal Tolerance Proteins in Tobacco Under Heavy Metal Toxicity

**DOI:** 10.3389/fgene.2019.00345

**Published:** 2019-04-24

**Authors:** Jikai Liu, Yongfeng Gao, Yunlai Tang, Dan Wang, XiaoMing Chen, Yinan Yao, Yaoling Guo

**Affiliations:** ^1^School of Life Sciences and Engineering, Southwest University of Science and Technology, Mianyang, China; ^2^State Defense Key Laboratory of the Nuclear Waste and Environmental Security, Southwest University of Science and Technology, Mianyang, China

**Keywords:** metal tolerance protein, evolution, expression, heavy metal, tobacco

## Abstract

Plant metal tolerance proteins (MTPs) comprise a family of membrane divalent cation transporters that play essential roles in plant mineral nutrition maintenance and heavy metal stresses resistance. However, the evolutionary relationships and biological functions of *MTP* family in tobacco remain unclear. In the present study, 26, 13, and 12 *MTPs* in three main *Nicotiana* species (*N. tabacum*, *N. sylvestris*, and *N. tomentosiformis*) were identified and designated, respectively. The phylogenetic relationships, gene structures, chromosome distributions, conserved motifs, and domains of NtMTPs were systematic analyzed. According to the phylogenetic features, 26 NtMTPs were classified into three major substrate-specific groups that were Zn-cation diffusion facilitators (CDFs), Zn/Fe-CDFs, and Mn-CDFs, and seven primary groups (1, 5, 6, 7, 8, 9, and 12). All of the NtMTPs contained a modified signature sequence and the cation_efflux domain, whereas some of them also harbored the ZT_dimer. Evolutionary analysis showed that *NtMTP* family of *N. tabacum* originated from its parental genome of *N. sylvestris* and *N. tomentosiformis*, and further underwent gene loss and expanded via one segmental duplication event. Moreover, the prediction of *cis*-acting elements (CREs) and the microRNA target sites of *NtMTP* genes suggested the diverse and complex regulatory mechanisms that control *NtMTPs* gene expression. Expression profile analysis derived from transcriptome data and quantitative real-time reverse transcription-PCR (qRT-PCR) analysis showed that the tissue expression patterns of *NtMTPs* in the same group were similar but varied among groups. Besides, under heavy metal toxicity, *NtMTP* genes exhibited various responses in either tobacco leaves or roots. 19 and 15 *NtMTPs* were found to response to at least one metal ion treatment in leaves and roots, respectively. In addition, NtMTP8.1, NtMTP8.4, and NtMTP11.1 exhibited Mn transport abilities in yeast cells. These results provided a perspective on the evolution of *MTP* genes in tobacco and were helpful for further functional characterization of *NtMTP* genes.

## Introduction

Metal ions such as Zinc (Zn), Cobalt (Co), Iron (Fe), Manganese (Mn), and Copper (Cu), which constitute essential trace elements in plants, have vital biological functions at low levels but can cause toxic effects at excessive amounts (Kolaj-Robin et al., 2015). Meanwhile, other non-essential elements, including cadmium, silver, lead and mercury, can also be absorbed and be toxic even at very low concentrations (Clemens, 2001). Correspondingly, plants have evolved a comprehensive network of metal uptake, efflux, chelation, trafficking, and storage mechanisms to ensure the precise metal homeostasis (Montanini et al., 2007). Specific transporters belonging to different protein families have been shown to play an important role in these regulatory processes.

Members of the cation diffusion facilitator (CDF) family are integral membrane divalent cation transporters that are involved in metal ions efflux from the cytoplasm either to the outside of the cell or into subcellular compartments (Gustin et al., 2011). Since their first identification in 1995 (Nies and Silver, 1995), CDF transporters have been widely identified in archaea, eubacteria and eukaryotes, and can be classified into three major groups (Zn-CDF, Fe/Zn-CDF, and Mn-CDF), based on the hypothesized or confirmed transported substrate specificities (Montanini et al., 2007). Sequence analyses showed that most of the CDF proteins possess six putative transmembrane spanners (Paulsen and Saier, 1997), a modified signature sequence between TMDs I and II (Paulsen and Saier, 1997; Montanini et al., 2007), and a characteristic C-terminal cation_efflux domain (PF01545).

In plants, CDF transporters are designated as metal-tolerance proteins (MTPs), and form seven groups (1, 5, 6, 7, 8, 9, and 12) according to the results of the phylogenetic analysis and annotation of *Arabidopsis* MTPs (Gustin et al., 2011). There were 12 and 10 *MTP* genes identified in *Arabidopsis* and rice genome, respectively, but only a few of them have been functionally characterized in detail. Zinc *Arabidopsis* transporter (ZAT), also called AtMTP1, was the first identified MTP protein (van der Zaal et al., 1999). Previous studies found that both AtMTP1 and AtMTP3 localized in the tonoplast and were involved in the Zn and/or Co tolerance by sequestering excess Zn^2+^ and/or Co^2+^ into the vacuole (Kobae et al., 2004; Desbrosses-Fonrouge et al., 2005; Arrivault et al., 2006; Kawachi et al., 2008). OsMTP1, a bivalent cation transporter localized in vacuole, was necessary for the Zn, Cd, Co and Fe translocation and ion homeostasis in rice (Yuan et al., 2012; Menguer et al., 2013). AtMTP5 and AtMTP12, another two Zn-CDF proteins, were found to form a functional complex to transport Zn into the Golgi (Fujiwara et al., 2015). There were four AtMTP proteins (AtMTP8-11) belonging to the Mn-CDF family. Among them, both AtMTP8 and AtMTP11 functioned as an Mn transporter that protects plant cells from Mn toxicity, and AtMTP8 was also involved in Mn and Fe localization in seeds (Delhaize et al., 2007; Eroglu et al., 2016; Chu et al., 2017). Rice harbored five Mn-CDF members (OsMTP8.1, OsMTP8.2, OsMTP9, OsMTP11, and OsMTP11.1). Both OsMTP8.1 and OsMTP8.2 were tonoplast-localized Mn transporters, and OsMTP9 was involved in efficient root Mn uptake (Chen et al., 2013; Ueno et al., 2015; Takemoto et al., 2017; Tsunemitsu et al., 2018b). Moreover, OsMTP11 played a crucial role in Mn tolerance through intracellular Mn compartmentalization, although the correct localization of this protein was still under debate (Farthing et al., 2017; Zhang and Liu, 2017; Ma et al., 2018; Tsunemitsu et al., 2018a). In addition, some MTP proteins from cucumber were recently isolated and their corresponding substrates were also specified. For example, CsMTP1 and CsMTP4 functioned as vacuole-localized Zn and Cd transporters (Migocka et al., 2014). CsMTP7 was a highly specific mitochondrial Fe importer (Migocka et al., 2018a). CsMTP8 was located in the vacuolar membrane and participated in the maintenance of Mn homeostasis (Migocka et al., 2014). CsMTP9 was found to be a plasma membrane H^+^-coupled Mn^2+^ and Cd^2+^ antiporter (Migocka et al., 2015b).

As the genome sequences become available for more species, a number of MTP proteins have been genome-widely identified in several plants species, including *Vitis vinifera*, *Brachypodium diastychon*, *Zea mays*, *Sorghum bicolor*, *Populus trichocarpa*, *Brassica rapa*, *Triticum aestivum*, and *Citrus sinensis* (Montanini et al., 2007; Gustin et al., 2011; Migocka et al., 2014; Fu et al., 2017; Vatansever et al., 2017; Li et al., 2018). Tobacco (*Nicotiana tabacum*) is one of the most widely cultivated non-food crops worldwide and is also an important model plant organism for molecular plant biological research (Sierro et al., 2013; Edwards et al., 2017). Like many other flowering plants, *N. tabacum* is an alloteraploid (2n = 4x = 48) with a large genome of approximately 4.5 Gb, which originated through the hybridization of the ancestral parents *N. sylvestris* (2n = 24) and *N. tomentosiformis* (2n = 24) (Leitch et al., 2008). However, due to the limit of genome sequence information, few of the MTP proteins in tobacco have been well characterized until now. In recent years, efforts have been conducted to decipher the genomes of this model and commercially important species (Sierro et al., 2013; Edwards et al., 2017), and the completion of high quality draft genomes provided an opportunity to perform a systematic analysis of tobacco *MTP* gene family at the genome-wide level. In this study, we successfully identified the *MTP* genes in three main *Nicotiana* species (*N. tabacum*, *N. sylvestris*, and *N. tomentosiformis*) and comprehensively analyzed their sequence and structural characteristics, as well as the evolutionary relationships. Besides, the *cis*-acting regulatory element distributions, and the potential microRNA target sites in *NtMTP* genes were further predicted. In addition, the expression profiles of *NtMTP* genes in different tobacco tissues and in response to heavy metal toxicity were also investigated. In the end, the metal transport abilities of six representative NtMTPs in yeast mutant cells were investigated. Results in this study would provide a basis for the isolation and functional characterization of *NtMTP* genes in future studies.

## Materials and Methods

### Identification of *MTP* Genes in Three *Nicotiana* Species

To identify the *MTP* genes in tobacco, the protein sequences of 12 MTPs in *Arabidopsis* which were obtained from TAIR10^1^ were used as queries in TBLASTN search against the genomes of *N. tabacum* (Nitab v4.5 cDNA Edwards et al., 2017), *N. sylvestris* and *N. tomentosiformis* at Sol Genomics Network^2^ with default parameters. After removing the redundant sequences manually, the non-redundant sequences were examined with InterProScan (Finn et al., 2017^3^), and the candidates containing any of the typical domains of MTP proteins were recognized as MTP proteins.

### Sequence Alignment and Phylogenetic Analysis

The sequence similarity of MTPs proteins between *N. tabacum* and *A. thaliana* were analyzed in blastp suite – 2 sequences program at National Center for Biotechnology Information (NCBI^4^). Each protein sequence of MTPs in *Arabidopsis* was used as the query sequence, and all 26 NtMTP protein sequences were used as the subject sequence.

For phylogenetic analysis, multiple sequence alignments at amino acid level were performed by ClustalW, and MEGA 6.0 software was used for phylogenetic tree construction by the Maximum Likelihood method based on the Jones-Taylor-Thornton (JTT) matrix-base model with bootstrap of 1000 replicates (Felsenstein, 1985; Jones et al., 1992; Tamura et al., 2013). The sequences of MTPs from *Vitis vinifera*, *Brachypodium diastychon*, *Zea mays*, *Oryza sativa*, *Sorghum bicolor*, and *Populus trichocarpa* were downloaded from the corresponding database as described by Migocka et al. (2014). The MTPs sequences from *Cucumis sativus* were retrieved from the GenBank database with the accession numbers of NP_001295856.1 (CsMTP1), AFJ24701.1 (CsMTP4), APM86800.1 (CsMTP5), APM86801.1 (CsMPT6), APM86799.1 (CsMPT7), AFJ24703.1 (CsMTP8), AFJ24702.1 (CsMTP9), XP_004147705.1 (CsMTP11), and APM86802.1 (CsMTP12).

### Physicochemical Parameters and Structure Characteristics of NtMTP Proteins

Molecular weight (MW), theoretical isoelectric pint (pI) and grand average of hydropathicity (GRAVY), were calculated using ProParam tool (Gasteiger et al., 2005^5^). Sub-cellular localizations were predicted by Plant-mPLoc server (Chou and Shen, 2010^6^). The putative transmembrane regions in NtMTP proteins were predicted by using the TMHMM Server V. 2.0 (Sonnhammer et al., 1998; Krogh et al., 2001^7^). Conserved motifs and domains in NtMTP sequences were predicted by the MEME program and the Pfam tool, respectively (Bailey et al., 2009^8^; Finn et al., 2016^9^).

### Gene Structure, Chromosomal Distribution, Gene Duplication, and Ka/Ks Analysis

The exon/intron structures, and chromosomal distributions of *NtMTP* genes were determined according to the genome annotation files at Sol Genomics Network^10^. Gene duplication events were analyzed by using Multiple Collinearity Scan toolkit (MCScanX) with the default parameters (Wang et al., 2012). Finally, the diagrams of exon/intron organization, protein structure, chromosomal location and gene duplication event were drawn by TBtools software (Chen et al., 2018^11^). The number of synonymous (Ks) and non-synonymous (Ka) substitutions per site of duplicated gene pair were calculated by DnaSP v6 (Rozas et al., 2017).

### *Cis*-Acting Regulatory Elements and miRNA Target Sites Prediction

The promoter sequences (up-stream 1000 bp) of *NtMTP* genes were retrieved from *N. tabacum* genomes database (Nitab v4.5 Genome Scaffolds Edwards et al., 2017) at Sol Genomics Network^12^. The obtained sequences were then uploaded in PlantCARE database for *cis*-acting regulatory elements analysis (Rombauts et al., 1999^13^). The coding sequences of *NtMTP* genes were analyzed by psRNATarget server for miRNA target sites prediction (Dai et al., 2018^14^).

### Transcriptome Data Analysis

To investigate the tissue expression patterns of *NtMTP* genes, the Illumina RNA-sequencing data of *N. tabacum* TN90 were downloaded from GenBank Sequence Read Archive (SRA) with the accession code SRP029183 and analyzed. The fragments per kilobase per million reads (FPKM) were calculated and log_2_ transformed to estimate the expression levels of *NtMTP* genes in eight different tobacco tissues. The resulting values were used to generate a heat map by TBtools software (Chen et al., 2018^15^).

### Plant Growth and Heavy Metal Treatments

Tobacco plants (variety K326) were grown hydroponically with half-strength Hoagland solution (pH 6.0) in the greenhouse with a 16:8 h light:dark cycle under a temperature of 24°C at day, 18°C at night. The nutrient solutions were renewed every 4 days and were continuously aerated and exchanged every 4 h per day. Four weeks old plants were transferred in nutrient solutions containing 0.5 M ZnSO_4_, 1 M MnSO_4_, 0.1 M CoCl_2_, 0.1 M CdCl_2_, 0.5 M FeSO_4_-EDTA, and 1 M MgSO_4_, respectively, and those grown in nutrient solution without any heavy metal supplied were regarded as control (CK). Twelve tobacco plants were used for each treatment. After 24 h of treatments, the leaves and roots of the plants were harvested separately and were immediately frozen in liquid nitrogen, and stored at -70°C for RNA extraction.

### RNA Extraction and qRT-PCR

Total RNAs were extracted and treated with DNase I to degrade any residual genomic DNA contamination using the RNAprep pure Plant Kit (TIANGEN, China) according to the manufacturer’s instructions. The purity and concentration of total RNA was estimated by micro volume spectrophotometer Q6000 (Quawell, United States), the quality and integrity of which was assessed by 1% (w/v) agarose gel analysis. After that, 2 μg of total RNA was reverse transcribed into cDNA using ReverTra Ace qPCR RT Kit (TOYOBO, Japan).

qRT-PCR was performed with the TransStart Green qPCR SuperMix (TransGen Biothech, China) using the CFX96 Real-Time System (Bio-Rad, United States). All the primers used for qRT-PCR analysis are presented in Supplementary Table S1. Five house-keeping genes, *NtL25* (GenBank accession L18908.1), *Ntubc2* (GenBank accession AB026056.1), *NtEF-1*α (GenBank accession AF120093.1), *NtRL2* (GenBank accession X62500.1), and *NtCYP1* (GenBank accession AY368274.1), were chosen as internal reference gene candidates for qRT-PCR. The geNorm v. 3.5 (Vandesompele et al., 2002) was used to evaluate the stability of these five internal reference genes. The two most stable reference genes, *NtL25* and *Ntubc2*, were used as internal reference. The qRT-PCR conditions were as follows: 95°C for 3 min, 39 cycles of 95°C for 10 s, 60°C for 30 s, followed by a melting curve protocol. Each experiment was performed with three technical replicates. The relative expression values were determined against the CK sample using the 2^– △ △ Ct^ method (Livak and Schmittgen, 2001).

### Plasmid Construction, Yeast Transformation, and Growth

To generate the yeast expression constructs, the cDNA of the leaves of control plants obtained as above was used as the template to amplify the full coding regions of six *NtMTP* genes by PCR using specific primers list in Supplementary Table S2. The PCR products were then cloned into the *Kpn*I and *Xba*I or *Kpn*I and *Eco*RI sites of pYES2 to yield recombinant plasmids pYES2-NtMTP1.2, pYES2-NtMTP5.2, pYES2-NtMTP7.2, pYES2-NtMTP8.1, pYES2-NtMTP8.4, and pYES2-NtMTP11.1, respectively.

The *Saccharomyces cerevisiae* strain BY4741 and five deletion mutants Y00829 (*zrc1*△), Y04534 (*pmr1*△), Y01613 (*cot1*△), Y04069 (*ycf1*△), and Y04169 (*ccc1*△) were obtained from the Euroscarf^16^. The plasmids were introduced into yeast by using the LiOAc/PEG method (Gietz and Schiestl, 2007). Yeast growth and metal sensitivity tests were performed as described previously with minor modifications (Migocka et al., 2015b). Briefly, transformed yeasts were grown in liquid synthetic complete medium supplemented with amino acids (-Uracil) and glucose (SC-U/Glu) overnight. Then the yeast cultures were resuspended in sterile deionized water and adjusted to OD600 = 0.2. 2 μL of serial dilutions were plated on solid SC-U/Glu medium without extra metal (control) and galactose-inducing SC-U medium (SC-U/Gal) supplemented with different heavy metals as indicated in the figures. Plates were incubated at 30°C for 2–4 days and photographed.

## Results

### Identification, Phylogeny, and Classification of *MTP* Genes in Tobacco

**Figure 1:**
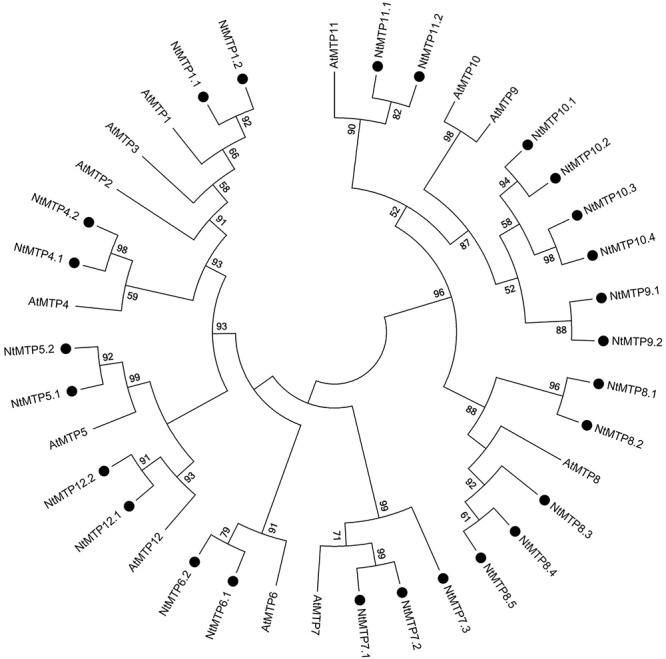
Phylogenetic relationship of MTP proteins in *N. tabacum* and *Arabidopsis*. The tree was generated using the MEGA 6.0 software by the Maximum Likelihood method based on the JTT matrix-base model with bootstrap of 1000 replicates. The black solid circles represent the MTP proteins from tobacco.

**Figure 2:**
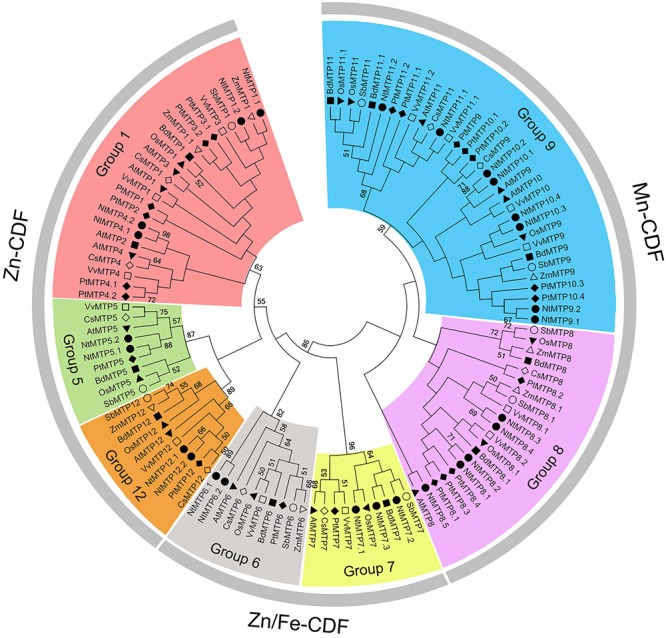
Phylogenetic relationship of MTP proteins in *N. tabacum* and other plant species. The tree was generated using the MEGA 6.0 software by the Maximum Likelihood method based on the JTT matrix-base model with bootstrap of 1000 replicates. 117 MTP proteins are clustered into three major substrate-specific groups and seven primary groups which are highlight in different colors. The solid squares represent the MTP proteins from Brachypodium. The reverse solid triangles represent the MTP proteins from Rice. The hollow circles represent the MTP proteins from sorghum. The solid circles represent the MTP proteins from tobacco. The solid diamonds represent the MTP proteins from poplar. The hollow squares represent the MTP proteins from grape. The solid triangles represent the MTP proteins from *Arabidopsis*. The hollow diamonds represent the MTP proteins from cucumber. The hollow triangles represent the MTP proteins from maize.

By using 12 AtMTP protein sequences as the queries, a total of 26 *NtMTP* genes were identified in *N. tabacum* genome. The sequence similarity and the phylogenetic relationship of the MTP proteins between *N. tabacum* and *A. thaliana* were further investigated. Based on the sequence identity and cover values, as well as the orthologous relationship, the 26 NtMTP proteins were designated as NtMTP1.1 to NtMTP12.2 (Figure 1, Supplementary Table S3, and Table 1). For each AtMTP protein, there were at least two MTP homologs in *N. tabacum* except for AtMTP2 and AtMTP3, where no corresponding NtMTP was found (Figure 1). To better understand the evolutionary relationships of *MTP* gene family members between tobacco and other plants, 117 MTP protein sequences from nine representative species, including four monocots (Brachypodium, rice, sorghum and maize) and five dicots (tobacco, *Arabidopsis*, cucumber, poplar and grape), were comprehensively analyzed and a phylogenetic tree was constructed. According to the classification of previous studies (Montanini et al., 2007; Gustin et al., 2011), the 117 plant MTP protein members were divided into three major substrate-specific groups (Zn-CDFs, Zn/Fe-CDFs, and Mn-CDFs) and seven primary groups (1, 5, 6, 7, 8, 9, and 12; Figure 2). Of the seven groups, group 9 makes the largest group containing 8 NtMTPs, while groups 5, 6, and 12 are the smallest groups with two NtMTPs each. There are three, four and five NtMTP members in groups 7, 1, and 8, respectively (Figure 2).

**Table 1:** Detail information of 26 *NtMTP* genes identified in current study.

				CDS	Protein
				length	size	MW			Sub-cellular
Gene name	Gene ID	Chromosome location	Strand	(bp)	(aa)	(kDa)	pI	GRAVY	localization	TMD number
*NtMTP1.1*	Nitab4.5_0001151g0150	Nt18:102607421..102611281	-	1059	352	38.905	6.1	0.157	Vacuole	5/out → in
*NtMTP1.2*	Nitab4.5_0002988g0010	Nitab4.5_0002988:256078..257334	-	1257	418	46.211	6	-0.002	Vacuole	6/in → in
*NtMTP4.1*	Nitab4.5_0004076g0010	Nt08:59691709..59693903	+	1230	409	45.909	6.15	0.219	Vacuole	6/in → in
*NtMTP4.2*	Nitab4.5_0000839g0150	Nt22:50409642..50410754	-	1113	370	41.539	6.19	0.212	Vacuole	6/in → in
*NtMTP5.1*	Nitab4.5_0001137g0070	Nt04:123742211..123752361	-	1182	393	44.149	6.31	0.072	Vacuole	5/in → out
*NtMTP5.2*	Nitab4.5_0004458g0020	Nitab4.5_0004458:178277..190151	-	1155	384	43.31	7.21	0.06	Vacuole	5/in → out
*NtMTP6.1*	Nitab4.5_0001447g0040	Nt22:117500587..117522354	+	4296	1431	158.06	6.4	-0.482	Nucleus/ Vacuole	0
*NtMTP6.2*	Nitab4.5_0001697g0140	Nt19:66326130..66347238	-	4653	1550	171.021	6.48	-0.36	Nucleus/ Vacuole	0
*NtMTP7.1*	Nitab4.5_0002661g0120	Nt22:18800780..18805755	+	1254	417	45.493	6.76	0.019	Vacuole	2/out → out
*NtMTP7.2*	Nitab4.5_0003757g0030	Nt22:16543160..16548333	-	1326	441	48.149	5.79	-0.02	Vacuole	4/in → in
*NtMTP7.3*	Nitab4.5_0012506g0020	Nitab4.5_0012506:602..3679	-	999	332	35.883	5.75	0.24	Vacuole	2/in → in
*NtMTP8.1*	Nitab4.5_0007829g0010	Nt06:19450246..19453636	+	1167	388	43.65	4.95	0.03	Vacuole	4/out → out
*NtMTP8.2*	Nitab4.5_0000009g0270	Nt04:115235977..115239588	-	1197	398	44.732	5.06	0.083	Vacuole	5/in → out
*NtMTP8.3*	Nitab4.5_0003555g0050	Nitab4.5_0003555:179266..183052	+	1329	442	49.788	5.59	0.095	Vacuole	5/in → out
*NtMTP8.4*	Nitab4.5_0005477g0050	Nitab4.5_0005477:33758..37539	+	1083	360	40.597	5.83	-0.003	Vacuole	4/out → out
*NtMTP8.5*	Nitab4.5_0001568g0050	Nitab4.5_0001568:22593..25965	-	891	296	32.808	6.13	0.067	C. membr./ Vacuole	3/out → in
*NtMTP9.1*	Nitab4.5_0003134g0040	Nt23:127076871..127080967	+	1206	401	45.459	7.28	-0.042	Vacuole	6/in → in
*NtMTP9.2*	Nitab4.5_0011518g0030	Nitab4.5_0011518:5351..11251	-	1080	359	41.138	8.64	-0.121	Vacuole	5/out → in
*NtMTP10.1*	Nitab4.5_0000598g0050	Nitab4.5_0000598:386608..392550	-	1263	420	48.258	6.44	-0.001	C. membr./ Vacuole	5/in → out
*NtMTP10.2*	Nitab4.5_0000388g0070	Nt24:64996534..65019815	-	1113	370	42.74	6.33	-0.236	Vacuole	3/in → out
*NtMTP10.3*	Nitab4.5_0008957g0050	Nitab4.5_0008957:741..3706	-	1170	389	44.438	6.44	-0.175	Vacuole	3/in → out
*NtMTP10.4*	Nitab4.5_0008908g0010	Nitab4.5_0008908:2840..9939	+	1152	383	43.301	6.47	-0.038	C. membr./ Vacuole	5/in → out
*NtMTP11.1*	Nitab4.5_0006851g0030	Nitab4.5_0006851:79070..84337	-	1119	372	42.406	4.99	-0.086	Vacuole	2/out → out
*NtMTP11.2*	Nitab4.5_0000914g0320	Nt13:17710444..17715754	-	1119	372	42.425	4.95	-0.081	Vacuole	3/in → out
*NtMTP12.1*	Nitab4.5_0001727g0060	Nt17:134884186..134886249	+	2064	687	76.997	6.29	-0.044	Vacuole	10/in → in
*NtMTP12.2*	Nitab4.5_0003431g0010	Nitab4.5_0003431:148091..149887	+	1797	598	66.899	6.2	-0.195	Vacuole	8/in → in

The characteristics of the *NtMTP* genes were analyzed in detail. The length of CDS sequence of *NtMTP* genes ranged from 891 bp (NtMTP8.5) to 4653 bp (NtMTP6.2), while the length of their encoded protein ranged from 296 to 1550 amino acid, and the relative MW ranged from 32.808 to 171.021 kDa, respectively (Table 1). Most of the NtMTP proteins have relatively low isoelectric points (pI < 7), except for NtMTP5.2, NtMTP9.1, and NtMTP9.2, which have a pI of 7.21, 7.28, and 8.64, respectively (Table 1). The GRAVY of the NtMTPs ranged from -0.482 (NtMTP6.1) to 0.24 (NtMTP7.3) (Table 1). Sub-cellular localization prediction results showed that all the NtMTP proteins localized to vacuole, with the dual localization predictions for NtMTP6.1 and NtMTP6.2 (nucleus or vacuole), and for NtMTP8.5, NtMTP10.1, and NtMTP10.4 (cellular membrane or vacuole) (Table 1). Notably, although more than half of the NtMTP proteins harbored four to six putative transmembrane domains (TMDs), NtMTP7.1, NtMTP7.3, and NtMTP11.1 had only two TMDs, NtMTP8.5, NtMTP10.2, NtMTP10.3, and NtMTP11.2 contained three TMDs, and NtMTP12.2 and NtMTP12.1 carried eight and ten TMDs, respectively. Particularly, NtMTP6.1 and NtMTP6.2 proteins lacked any of the TMDs (Table 1).

### Gene Structure Analysis and Chromosomal Localization of *NtMTP* Genes

**Figure 3:**
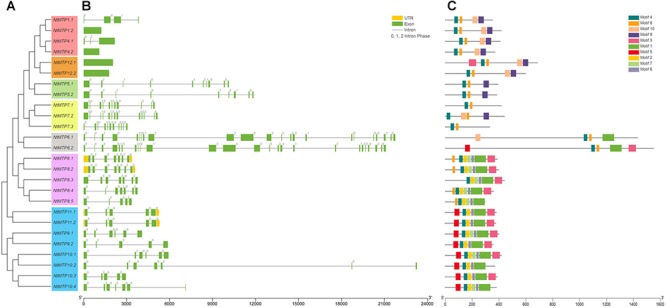
Phylogenetic relationships, gene structure and conserved motifs in *MTP* genes from *N. tabacum*. **(A)** Phylogenetic tree was constructed using the MEGA 6.0 software based on the full-length sequences of tobacco MTP proteins. Seven primary groups are shown in different colors. **(B)** Exon-intron structure of tobacco *MTP* genes. Yellow boxes indicated untranslated 5′- and 3′-regions; green boxes indicate exons; black lines indicate introns. The number indicates the phases of corresponding introns. **(C)** Conserved motifs were identified by MEME and displayed in different colored boxes.

To gain more insight into the evolution of the *MTP* gene family in *N. tabacum*, the intron-exon structures of *NtMTP* genes were examined. As shown in Figure 3A,B, *NtMTP* genes that clustered closely showed similar exon numbers and intron phases, which was consistent with the results of phylogenetic analysis and classification mentioned above. Zn-CDFs contained the smallest number of exons (group 1 contained 1–4 exons, group 12 contained only one exon), except for group 5 which possessed 9–10 exons, while Zn/Fe-CDFs comprised the highest number of exons (group 6 contained 23 or 25 exons, group 7 contained 11–13 exons) (Figure 3A,B). Two groups (group 8 and group 9) from Mn-CDFs contained the same range of exon numbers (5–7) (Figure 3A,B). In addition, phase 0 and phase 2 introns were widely distributed among all of the *NtMTP* genes, while phase 1 intron was only observed in members of group 1, group 5, group 6, and group 7 (Figure 3A,B).

**Figure 4:**
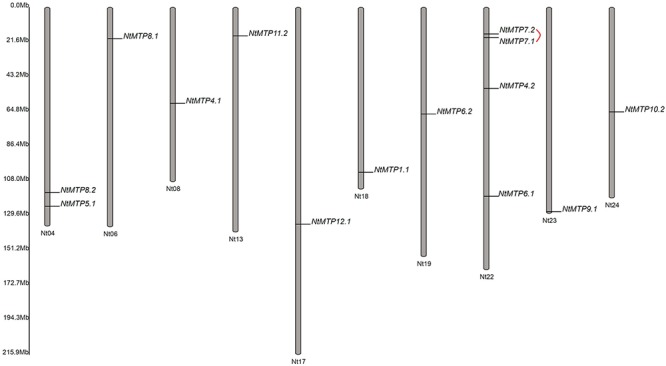
The distributions of *NtMTP* genes on *N. tabacum* chromosome. Chromosome number was indicated at the bottom of each chromosome, and the size of which was labeled on the left of the figure. Segmental duplicated gene pair was linked with red line.

Based on the physical location information from the database of *N. tabacum* genome, the chromosomal localizations of *NtMTP* genes were determined. 14 of the 26 *NtMTP* genes were located in 10 out of the 24 tobacco chromosomes (Figure 4). Chromosomes Nt06, Nt08, Nt13, Nt17, Nt18, Nt19, Nt23, and Nt24 contained only one *NtMTP* gene, while chromosome Nt04 and Nt22 carried two and four *NtMTP* genes, respectively. However, none of the *NtMTP* genes were mapped onto chromosomes Nt01, Nt02, Nt03, Nt05, Nt07, Nt09, Nt10, Nt11, Nt12, Nt14, Nt15, Nt16, Nt20, and Nt21 (Figure 4). Besides, since the complete genome sequence of *N. tabacum* was not yet built, there were still 12 *NtMTP* genes that could not map onto any chromosome. Nevertheless, these results would be valuable for future investigation of the evolutionary process of *NtMTP* genes.

### Multiple Sequence Alignment, Conserved Motifs, and Domain Architectures in *NtMTP*-Encoded Proteins

By analyzing the phylogeny of 273 representative CDF proteins, Montanini et al. identified a modified signature existing in the trans-membrane regions of the gene family members, and suggested a functional role of the conserved group-residues in metal selectivity (Montanini et al., 2007). Moreover, the consensus residues HxxxD (x = any amino acid) and DxxxD were identified to represent the sequence characteristics of both of Zn-CDFs and Fe/Zn-CDFs, and Mn-CDFs, respectively (Montanini et al., 2007). To explore the sequence features of the NtMTP proteins, the amino acid sequences of the AtMTPs and NtMTPs from three substrate-specific groups were multiple aligned by ClustalX, respectively. Results showed that all the AtMTP and NtMTP proteins carried a conserved signature consisting of 44 amino acids at the N terminus. In addition, there were two and one conserved HxxxD residues in Zn-CDFs and Zn/Fe-CDFs, respectively, and two DxxxD residues were found in the Mn-CDF subgroups (Supplementary Figure S1).

To gain further insight into the structure characteristics of the NtMTP proteins, their amino acid sequences were submitted to MEME program for conserved motif analysis. As shown in Figure 3C, ten motifs were in total detected in NtMTP family members, whereas only six of them were found to encode functional domains when subjected to Pfam (Figure 3C and Supplementary Table S4). Motif 1, 4, 8, and 10 were annotated as cation_efflux, motif 3 was annotated as ZT_dimer (PF16916), while motif 9 was found to encode SpoIIIAC. Among the ten motifs, motif 4 was widely distributed in all of the 26 NtMTPs (Figure 3C). In addition, intragroup members usually harbored similar types and distribution of motifs. For example, all of the NtMTP proteins from Zn-CDFs harbored motif 4, 8, 9, and 10, except for NtMTP5.1 and NtMTP5.2 lacked motif 10, and NtMTP12.1 carried motif 3 in addition. Nearly all of the NtMTPs from group 8 contained motif 1, 2, 3, 4, 6, 7, and 8, except for NtMTP8.3 and NtMTP8.5. The motif composition in members of group 9 were similar to those of group 8, except for the fact that motif 5 was detected at the N terminus instead of motif 8 (Figure 3C).

**Figure 5:**
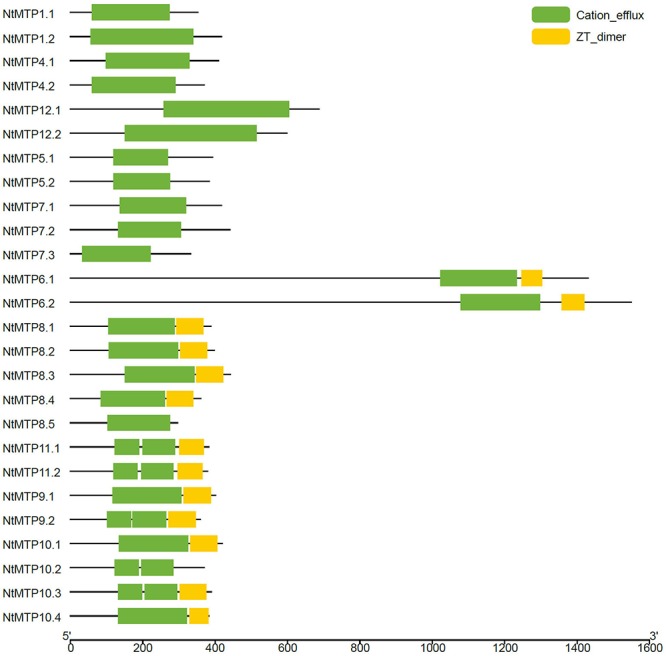
Distributions of the conserved domains in NtMTP proteins. Green boxes indicate cation_efflux domains; yellow boxes indicate ZT_dimers.

As described earlier, the cation_efflux domain was one of the typical features of MTP transporters. Hence, the domain architectures in NtMTP proteins were also analyzed. The results showed that the cation_efflux domain could be detected in all the NtMTP proteins, however, ZT_dimers, which are zinc transporter dimerization domains, were only detected in group 6, and in almost all the Mn-CDF members except for NtMTP8.5 and NtMTP10.2 (Figure 5).

### Evolutionary Analysis of *MTP* Genes in Three Main *Nicotiana* Species

**Figure 6:**
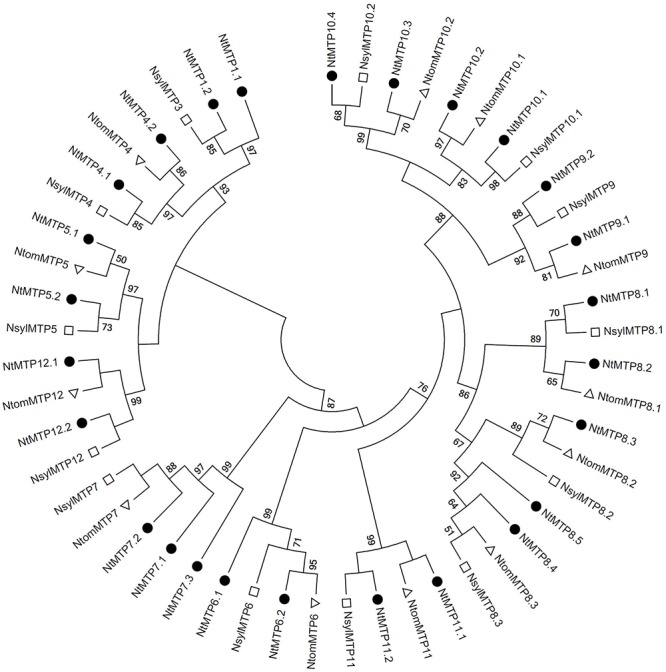
Phylogenetic relationship of MTP proteins in three main *Nicotiana* species. The tree was generated using the MEGA 6.0 software by the Maximum Likelihood method based on the JTT matrix-base model with bootstrap of 1000 replicates. The solid circles represent the MTP proteins from *N. tabacum*. The hollow squares represent the MTP proteins from *N. sylvestris*. The hollow triangles represent the MTP proteins from *N. tomentosiformis*.

As mentioned earlier, *N. tabacum* is an alloteraploid likely arising from hybridization of the ancestral parents *N. sylvestris* and *N. tomentosiformis*, and the *NtMTP* family had the largest member number than any other known plant in the MTP family, which prompted us to detect the retention or loss of *MTP* genes after polyploidization. By using the same method of identifying the *NtMTP* genes, 13 and 12 *MTP* genes were identified from *N. sylvestris* and *N. tomentosiformis* genomes, respectively (Supplementary Table S5), and the phylogenetic relationship of the MTP proteins among the three main *Nicotiana* species was further investigated. As exhibited in Figure 6, unlike most of the NtMTPs which have orthologs of their presumptive parents, NtMTP1.1 and members of group 7 had no clear orthologs from either of their parents, indicating that these genes may have originated after polyploidization, most likely from gene duplication events. In addition, both *N. sylvestris* and *N. tomentosiformis* genomes contained three MTPs of group 8 respectively, which were expected to produce six MTP8 paralogs in their progeny genome. However, *N. tabacum* genome actually carried only five corresponding members, indicating that one NtMTP gene in group 8 may be lost during the course of evolution.

To better understand the impact of gene duplication on the expansion of the *NtMTP* gene family, the possible tandem and segmental duplication events were analyzed by using BLASTP and MCScanX methods. Results showed that there was only one gene pair (*NtMTP7.1*/*NtMTP7.2*) found as segmental duplication event despite of their same chromosome location, however, no tandem duplication event was identified (Figure 4). Based on this result, the Ka/Ks ratio which is an indicator of selective pressure at the sequence level was further calculated. Ka/Ks < 1 means purifying selection or negative selection; Ka/Ks = 1 means neutral selection; Ka/Ks > 1 means positive selection (Hurst, 2002). The result showed that the Ka/Ks ratio of *NtMTP7.1*/*NtMTP7.2* was 0.5349 (<1), suggesting that this duplicated pair might undergo negative selection (Table 2).

**Table 2:** Ka/Ks analysis for *NtMTP* genes.

					Positive
Duplicated pair	Duplicate type	Ka	Ks	Ka/Ks	selection
NtMTP7.1/NtMTP7.2	Segmental	0.0399	0.0746	0.5349	No

*Ka/Ks < 1 means negative selection, Ka/Ks = 1 means neutral selection, and Ka/Ks > 1 means positive selection.*

### *Cis*-Acting Elements in the Promoter Regions of *NtMTP* Genes

The *cis*-acting elements (CREs) are regions of non-coding DNA which regulate the transcription of neighboring genes through binding by transcription factors and/or other regulatory molecules (Wittkopp and Kalay, 2011). To identify the CREs in the promoter regions, the 1000 bp upstream sequences of *NtMTP* genes were retrieved from the database of *N. tabacum* genome and analyzed using PlantCARE, except that 731, 898, and 506 bp upstream regions of *NtMTP5.2*, *NtMTP7.3*, and *NtMTP10.3* were analyzed due to the limitation of genomic sequences. As shown in Table 3 and Supplementary Table S6, a total of 2269 putative CREs were identified, including 1689 elements which were related to gene transcription, 252 elements associated to light responsiveness, 116 elements related to phytohormone responsiveness, 86 elements involved in abiotic stress responsiveness, 69 elements related to tissue expression, 24 elements related to circadian control, 20 elements related to biotic stress responsiveness, 10 elements related to site-binding and 3 elements related to secondary metabolism.

**Table 3:** Summary of the *cis*-acting regulatory elements identified in the promoter regions of *NtMTP* genes.

	Gene	Abiotic	Biotic	Tissue	Secondary	Phytohormonal	Light	Circadian
	transcription	stress	stress	expression	metabolism	response	response	control	Site-binding
*NtMTP1.1*	42	3	1	1	0	5	14	2	1
*NtMTP1.2*	38	6	1	5	0	8	6	1	1
*NtMTP4.1*	68	2	1	4	0	3	8	2	0
*NtMTP4.2*	64	3	0	3	0	7	8	1	0
*NtMTP5.1*	111	2	1	2	1	3	8	0	0
*NtMTP5.2*	95	0	1	1	1	2	4	1	0
*NtMTP6.1*	55	5	0	1	0	7	7	1	1
*NtMTP6.2*	48	2	0	2	0	3	8	0	0
*NtMTP7.1*	83	2	0	3	0	9	8	1	0
*NtMTP7.2*	76	2	0	1	0	4	14	0	1
*NtMTP7.3*	66	7	2	3	0	5	5	1	0
*NtMTP8.1*	51	4	1	3	0	6	12	1	0
*NtMTP8.2*	64	7	0	6	1	4	14	1	0
*NtMTP8.3*	74	5	0	0	0	3	13	1	0
*NtMTP8.4*	84	3	2	6	0	6	10	0	0
*NtMTP8.5*	68	6	0	0	0	5	13	0	0
*NtMTP9.1*	72	6	1	2	0	4	3	3	0
*NtMTP9.2*	63	1	1	5	0	5	6	2	2
*NtMTP10.1*	53	2	5	5	0	0	13	0	0
*NtMTP10.2*	63	6	1	4	0	4	18	1	0
*NtMTP10.3*	45	0	0	1	0	0	4	1	0
*NtMTP10.4*	96	3	1	3	0	2	4	1	2
*NtMTP11.1*	51	4	0	2	0	10	19	0	0
*NtMTP11.2*	87	1	1	2	0	7	16	1	2
*NtMTP12.1*	48	2	0	2	0	1	10	1	0
*NtMTP12.2*	24	2	0	2	0	3	7	1	0

Among these elements, CAAT-box and TATA-box, which were are common CREs, appeared to be the most abundance elements (with the number of 529 and 1146, respectively) and were commonly shared by all *NtMTP* genes. Besides, 33 different types of members were found in light responsiveness elements, such as Sp1, G-box, GT1-motif, Box 4 and G-Box, etc. Comparably, 12 types of elements were found in charge of six kinds of hormones, including ABRE and CE3 involved in abscisic acid (ABA) responsiveness, P-box, GARE-motif, TATC-box in gibberellin responsiveness, TCA-element and SARE in salicylic acid responsiveness, CGTCA-motif and TGACG-motif in jasmonic acid (MeJA) responsiveness, TGA-element and AuxRR-core in auxin responsiveness and ERE in ethylene responsiveness. Additionally, abiotic stress elements comprised LTR for low temperature responsiveness, MBS for drought inducibility, TC-rich repeat for defense/stress responsiveness, HSE for heat stress responsiveness, WUN-motif for wound responsiveness, ARE and GC-motif for anaerobic induction. Moreover, elements associated with tissue expression included CAT-box and CCGTCC-box for meristem expression and meristem specific activation, respectively, RY-element for seed-specific regulation, GCN4_motif and Skn-1_motif for endosperm expression, HD-Zip 1 for palisade mesophyll cells differentiation, HD-Zip 2 for leaf morphology development control and as-2-box for shoot-specific expression and light responsiveness. Notably, circadian involved in circadian control was distributed in the promoter regions of over half of the *NtMTP* genes. Whereas, elements involved in biotic stress responsiveness, secondary metabolism and site-binding were less abundant than others. Taken together, the presence of these elements indicated that *NtMTP* genes could be transcriptionally regulated by multiple stimuli, and participate in various plant metabolic processes.

### Potential MicroRNA Target Sites in *NtMTP* Genes

MicroRNAs (miRNAs) are small non-coding RNA molecules that can play important regulatory roles in gene expression by targeting mRNAs for cleavage or translational repression (Bartel, 2004). To give insights into the post-transcriptional regulation of the *NtMTP* genes, their potential miRNA target sites were searched using plant small RNA target analysis server (psRNATarget). With the expectation score lower than 4.0, in total eight NtmiRNAs comprising target sites in ten *NtMTP* genes were identified (Table 4). All three members of group 7 can be targeted by nta-miR6144, whereas *NtMTP7.1* can also be targeted by nta-miR1446. Moreover, *NtMTP4.1*, *NtMTP9.1*, and *NtMTP6.2* were targeted by nta-miR172j, nta-miR397 and nta-miR6020a-5p, respectively. Both *NtMTP8.1* and *NtMTP8.2* were targeted by nta-miR6019a and nta-miR6019b, and both *NtMTP10.3* and *NtMTP10.4* were targeted by nta-miR479a, respectively. Notably, except for nta-miR1446/*NtMTP7.3* and nta-miR172j/*NtMTP4.1*, most of the identified miRNA-targeted *NtMTP* genes were predicted to be silenced by cleavage inhibition. The accessibility of the mRNA target site to small RNA has been identified as one important factor involved in target recognition (Marín and Vanícek, 2010). The energy required to unpair the secondary structure around target site (UPE), which represented the target accessibility, was also calculated by RNAup (Mückstein et al., 2006). The results showed that the UPE varied from 10.78 (nta-miR479a/*NtMTP10.4*) to 22.218 (nta-miR6020a-5p/*NtMTP6.2*).

**Table 4:** The potential miRNA target sites in *NtMTP* genes.

				miRNA	
miRNA Acc.	Target Acc.	Expectation	UPE	length	Target start-end	miRNA aligned fragment	Target aligned fragment	Inhibition
nta-miR6144	*NtMTP7.1*	2	17.908	21	1124–1144	UGGCAACUUCUUCAUCAUGCC	UCUAUGGUGAGGAAGUUGUCA	Cleavage
nta-miR6144	*NtMTP7.2*	2	16.125	21	1196–1216	UGGCAACUUCUUCAUCAUGCC	UCUAUGGUGAGGAAGUUGUCA	Cleavage
nta-miR6144	*NtMTP7.3*	3	17.009	21	875–895	UGGCAACUUCUUCAUCAUGCC	AUUAUGGUGAAGAAGUUGUGA	Cleavage
nta-miR1446	*NtMTP7.3*	3.5	14.554	22	966–987	UGAACUCUCUCCCUCAAUGGCU	AGACAUUGAGGCACACAGUUCA	Translation
nta-miR172j	*NtMTP4.1*	3.5	19.11	21	115–135	GGAAUCUUGAUGAUGCUGCAU	AUGGAGCAACAUGAGGAUUCC	Translation
nta-miR479a	*NTMTP10.4*	3.5	10.78	22	541–562	CGUGAUAUUGGUUUGGCUCAUC	AAAAAUCCAAACCAGUAUCACU	Cleavage
nta-miR479a	*NtMTP10.3*	3.5	11.458	22	541–562	CGUGAUAUUGGUUUGGCUCAUC	AAAAAUCCAAACCAGUAUCACU	Cleavage
nta-miR397	*NtMTP9.1*	4	18.748	20	427–446	AUUGAGUGCAGCGUUGAUGU	GCAUCAACUUUGGACUCACU	Cleavage
nta-miR6019a	*NtMTP8.2*	4	13.42	22	789–810	UACAGGUGACUUGUAAAUGUUU	AAACAUUUACAAGUACCCUAUA	Cleavage
nta-miR6019a	*NtMTP8.1*	4	14.321	22	768–789	UACAGGUGACUUGUAAAUGUUU	AAACAUUUACAAGUACCCUAUA	Cleavage
nta-miR6019b	*NtMTP8.2*	4	13.42	22	789–810	UACAGGUGACUUGUAAAUGUUU	AAACAUUUACAAGUACCCUAUA	Cleavage
nta-miR6019b	*NtMTP8.1*	4	14.321	22	768–789	UACAGGUGACUUGUAAAUGUUU	AAACAUUUACAAGUACCCUAUA	Cleavage
nta-miR6020a-5p	*NtMTP6.2*	4	22.218	21	4059–4079	AAAUGUUUUUCGAGUAUCUUC	UGAGAUAUUUGAGAAACAUGG	Cleavage

### Expression Patterns of *NtMTP* Genes in Different Tissues Under Normal Conditions

**Figure 7:**
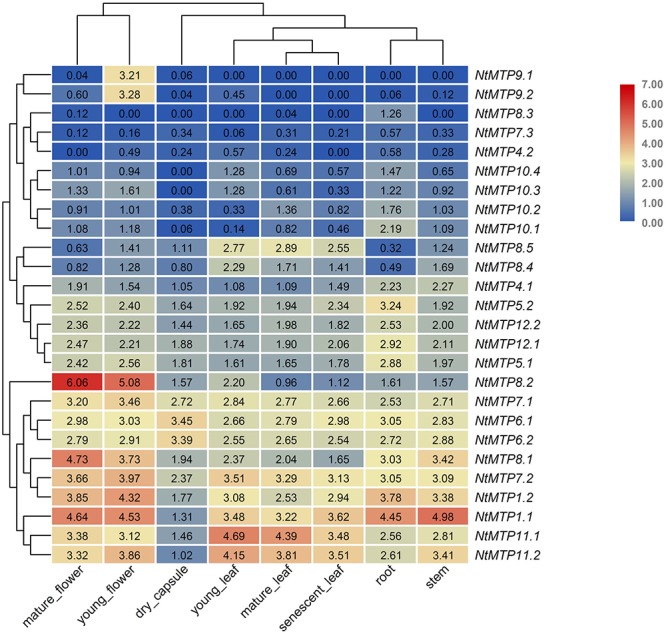
Heatmaps showing the abundance of *NtMTP* transcripts in different tobacco tissues at different growth stages. Normalized gene expression (FPKM) is expressed in log2 ratio.

The expression patterns of *NtMTP* genes in eight different tobacco tissues, including mature flower, young flower, dry capsule, young leaf, mature leaf, senescent leaf, root, and stem, were investigated using the Illumina RNA sequencing data from GenBank SRA (Sierro et al., 2014). As shown in Figure 7, the tissue expression patterns of *NtMTPs* among the seven groups were different, whereas those of members within each group were almost similar (Figure 7). *NtMTP1.1* and *NtMTP1.2*, which were group 1 members, had the highest expression in stem and mature flower, respectively, and the lowest expression in dry capsule. The expression levels of *NtMTP4.1* and *NtMTP4.2* were similar, although *NtMTP4.1* showed higher expression than *NtMTP4.2* in every tissues tested. Genes from groups 5 and 12 displayed constitutive expression in all tissues tested, and both had relatively higher expression levels in flower and root. Moreover, the expression levels of genes from both groups 6 and 7 were similar, except for *NtMTP7.3*, which was seldom expressed in all studied tissues. In addition, all gene members in group 8 showed tissue specific expression patterns. *NtMTP8.1* was highly expressed in flower, stem and root. *NtMTP8.2* was strongly expressed in both mature flower and young flower, and notably, the expression level in mature flower was the highest compared with other *NtMTPs* in tobacco tissues. Both *NtMTP8.4* and *NtMTP8.5* displayed abundant expression in three different types of leaf and stem, and low expression in root. However, *NtMTP8.3* gene was not expressed in most tissues but was only weakly expressed in root, mature flower and mature leaf. In group 9, *NtMTP9.1* and *NtMTP9.2* showed high expression in young flower, but weak or no expression in other tissues. All four members of *NtMTP10* showed weak expression in all tested tissues, except for *NtMTP10.1* in root. *NtMTP11.1* and *NtMTP11.2* exhibited abundant expression in all tissues, with relatively low expression in dry capsule.

### Expression Patterns of *NtMTP* Genes Under Heavy Metal Toxicity

To further explore the biological functions of MTP proteins in tobacco, 4 weeks old hydroponic tobacco plants were subjected to six different heavy metals, including five previously reported MTP protein transporting metal ions (Zn, Mn, Co, Cd, and Fe) and one representative macroelement Mg. The relative expression levels of *NtMTP* genes in response to these heavy metals in tobacco leaves and roots were investigated by qRT-PCR, respectively.

**Figure 8:**
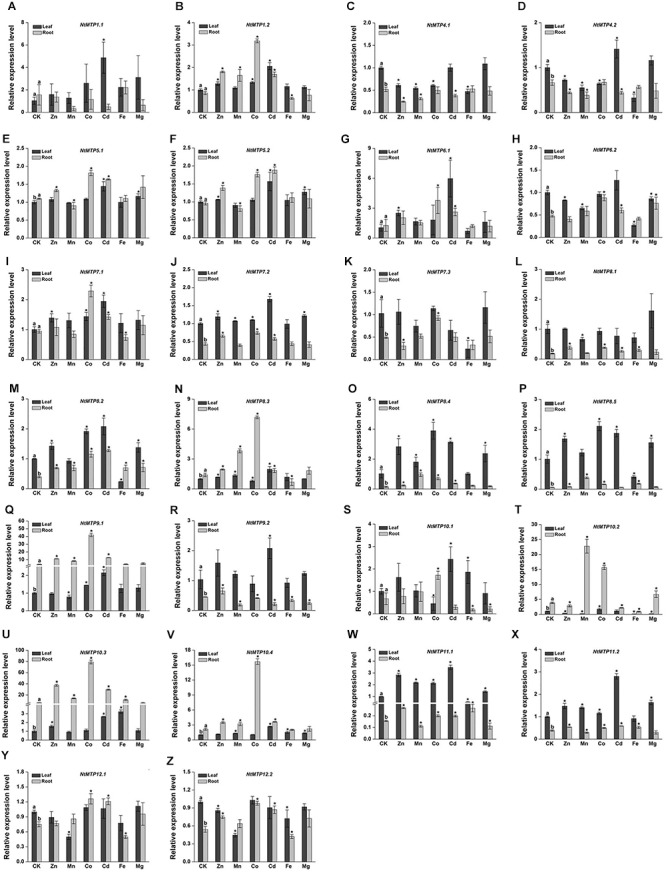
Expression levels of *NtMTPs* under different heavy metal treatments. Data represent means ( ± SD) of three biological replicates. CK represent control samples. Different letters (a and b) indicate significant differences between leaf and root under normal condition (*n* = 12, *P* < 0.05, Student’s *t*-test). Asterisks indicate significant differences between the treatment samples and the corresponding control samples in leaf or root. (*n* = 12, *P* < 0.05, Student’s *t*-test). **A–Z** stands for the NtMTP1.1-NtMTP12.2, respectively.

The differential tissue expression patterns of *NtMTP* genes under normal conditions (CK) were analyzed first. 14 *NtMTP* genes showed relative higher expression levels in leaves than in roots. The expressions of *NtMTP5.1*, *NtMTP8.3*, *NtMTP9.1*, *NtMTP10.2*, *NtMTP10.3*, and *NtMTP10.4* genes in the tobacco roots were higher than those in the leaves, whereas *NtMTP1.1*, *NtMTP1.2*, *NtMTP5.2*, *NtMTP6.1*, *NtMTP7.1*, and *NtMTP10.1* genes exhibited similar expression levels between these two tissues (Figure 8).

Under heavy metal toxicity, *NtMTP* genes exhibited various responses in either tobacco leaves or roots, and an overview of gene expression changes of *NtMTPs* was listed in Table 5. In leaves, all the *NtMTP* genes except *NtMTP5.1*, *NtMTP5.2*, *NtMTP7.1*, *NtMTP7.2*, *NtMTP8.1*, *NtMTP8.3*, and *NtMTP12.1* were responsive to at least one metal ion treatment. Twelve genes (*NtMTP1.1*, *NtMTP1.2*, *NtMTP6.1*, *NtMTP8.2*, *NtMTP8.4*, *NtMTP9.1*, *NtMTP9.2*, *NtMTP10.1*, *NtMTP10.3*, *NtMTP10.4*, *NtMTP11.1*, and *NtMTP11.2*) were up-regulated by Cd treatment. In contrast to Cd, other metal ions had diverse effects on the change of expression levels of *NtMTPs*. *NtMTP6.1*, *NtMTP8.4*, and *NtMTP11.1* were up-regulated by Zn, whereas *NtMTP10.2* was down-regulated; *NtMTP11.1* was increased by Mn, whereas *NtMTP10.2* and *NtMTP12.2* were repressed; *NtMTP8.4*, *NtMTP8.5*, and *NtMTP11.1* were up-regulated by Co, whereas *NtMTP10.1* were down-regulated; *NtMTP4.1*, *NtMTP4.2*, *NtMTP6.2*, *NtMTP7.3*, *NtMTP8.2*, and *NtMTP10.2* were repressed by Fe, whereas *NtMTP10.3* was increased; *NtMTP8.4* was up-regulated by Mg, whereas *NtMTP10.2* was down-regulated (Figure 8). In roots, half of the *NtMTP* genes were up-regulated by Co treatment, but under excess Mg condition, only *NtMTP10.1* was down-regulated. *NtMTP1.2*, *NtMTP8.1*, *NtMTP9.1*, and *NtMTP10.3* were up-regulated by Zn, whereas *NtMTP4.1* was down-regulated; *NtMTP8.3*, *NtMTP8.4*, *NtMTP8.5*, *NtMTP9.1*, *NtMTP10.2*, and *NtMTP10.3* were increased by Mn, only *NtMTP9.2* was repressed; *NtMTP6.1*, *NtMTP8.2*, *NtMTP8.4*, *NtMTP9.1*, and *NtMTP10.3* were up-regulated by Cd, whereas *NtMTP9.2* were down-regulated; *NtMTP8.3*, *NtMTP10.1* and *NtMTP10.2* were repressed by Fe, only *NtMTP8.5* was increased (Figure 8).

**Table 5:** Overview of *NtMTP* genes in response to different heavy metal stresses.

Gene name	In leaf						In root					
	Zn	Mn	Co	Cd	Fe	Mg	Zn	Mn	Co	Cd	Fe	Mg
*NtMTP1.1*	No	No	No	++	No	No	No	No	No	No	No	No
*NtMTP1.2*	No	No	No	+	No	No	+	No	+	No	No	No
*NtMTP4.1*	No	No	No	No	-	No	-	No	No	No	No	No
*NtMTP4.2*	No	No	No	No	-	No	No	No	No	No	No	No
*NtMTP5.1*	No	No	No	No	No	No	No	No	No	No	No	No
*NtMTP5.2*	No	No	No	No	No	No	No	No	No	No	No	No
*NtMTP6.1*	+	No	No	++	No	No	No	No	+	+	No	No
*NtMTP6.2*	No	No	No	No	-	No	No	No	No	No	No	No
*NtMTP7.1*	No	No	No	No	No	No	No	No	+	No	No	No
*NtMTP7.2*	No	No	No	No	No	No	No	No	No	No	No	No
*NtMTP7.3*	No	No	No	No	--	No	No	No	No	No	No	No
*NtMTP8.1*	No	No	No	No	No	No	+	No	+	No	No	No
*NtMTP8.2*	No	No	No	+	--	No	No	No	+	+	No	No
*NtMTP8.3*	No	No	No	No	No	No	No	+	++	No	-	No
*NtMTP8.4*	+	No	+	+	No	+	No	++	++	+	No	No
*NtMTP8.5*	No	No	+	No	No	No	No	++	+	No	+	No
*NtMTP9.1*	No	No	No	+	No	No	+	+	+++	+	No	No
*NtMTP9.2*	No	No	No	+	No	No	No	-	No	-	No	No
*NtMTP10.1*	No	No	-	+	No	No	No	No	+	No	-	-
*NtMTP10.2*	-	--	No	No	---	--	No	++	++	No	--	No
*NtMTP10.3*	No	No	No	+	+	No	++	+	+++	++	No	No
*NtMTP10.4*	No	No	No	+	No	No	No	No	++	No	No	No
*NtMTP11.1*	+	+	+	+	No	No	No	No	No	No	No	No
*NtMTP11.2*	No	No	No	+	No	No	No	No	No	No	No	No
*NtMTP12.1*	No	No	No	No	No	No	No	No	No	No	No	No
*NtMTP12.2*	No	-	No	No	No	No	No	No	No	No	No	No

*“+” and “-” means: 2 < change fold < 4; “++” and “--” means: 4 < change fold < 8; “+++” and “---” means: 8 < change fold < 16.*

### Effect of NtMTPs on the Metal-Sensitive Phenotypes of Yeast Mutants

**Figure 9:**
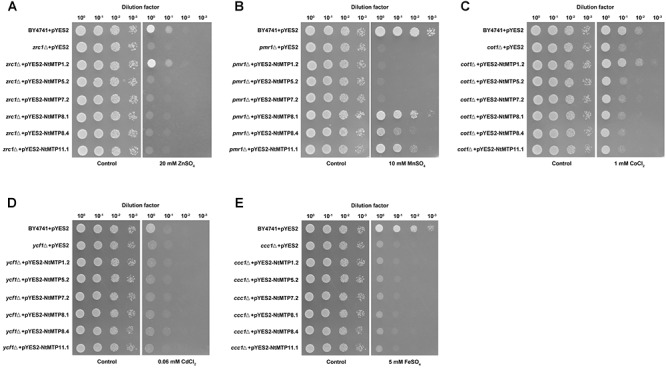
Complementation of yeast mutants on solid medium containing heavy metals. *S. cerevisiae* wild-type strain BY4741 was transformed with the empty vector pYES2, and mutants strains were transformed with the empty vector pYES2 or with the vectors carrying the *NtMTP* gene, respectively. Yeast cultures were adjusted to OD600 = 0.2, and 2 μL of serial dilutions (10-fold, from left to right in each panel) were spotted on SC-U/Gal medium supplemented with 20 mM ZnSO_4_ **(A)**, 10 mM MnSO_4_ **(B)**, 1 mM CoCl_2_ **(C)**, 60 μM CdCl_2_ **(D)**, or 5 mM FeSO_4_ **(E)** or on the SC-U/Glu medium (control) without the supplementation. The plates were incubated for 2–4 days at 30°C. The images are representative for three independent experiments.

Previous studies found that NtMTP1a and NtMTP1b from *N. tabacum* cv. *samsun* operated by sequestering Zn and Co into vacuoles to reduce the toxicity of these metals to yeast cell (Shingu et al., 2005). In order to better understand the metal selectivities of NtMTPs, a yeast metal sensitivity test assay was carried out by expressing six randomly selected genes (*NtMTP1.2*, *NtMTP5.2*, *NtMTP7.2*, *NtMTP8.1*, *NtMTP8.4*, and *NtMTP11.1*) in wild type yeast strain BY4741 and five deletion mutants which were deficient in various metal transporters. As shown in Figure 9, NtMTP1.2 clearly rescued the sensitivities of *zrc1*△ to Zn and *cot1*△ to Co. Also, the growth of *pmr1*△ in toxic Mn was restored by NtMTP8.1, NtMTP8.4, and NtMTP11.1, respectively. In contrast, the expression of neither *NtMTP5.2* nor *NtMTP7.2* complemented the sensitive phenotypes of any tested mutant strains grown in excess of different metals. These results suggested that NtMTP1.2 was a transporter of both Zn^2+^ and Co^2+^. On the other hand, NtMTP8.1, NtMTP8.4 and NtMTP11.1 could transport Mn^2+^ in yeast cell.

## Discussion

*MTP* genes encode membrane divalent cation transporters that participated in tolerating and transporting various heavy metals, and may play essential roles in plant mineral nutrition maintenance and resistance to stresses caused by metals (Clemens, 2001; Gustin et al., 2011; Ricachenevsky et al., 2013). In the present study, we successfully identified 26, 13, and 12 *MTP* genes in three main *Nicotiana* species (*N. tabacum*, *N. sylvestris*, and *N. tomentosiformi*s), respectively, and named these MTPs based on the sequence similarities and orthologous relationships between them and AtMTPs.

The phylogenetic relationships of the MTP proteins between *N. tabacum* and *Arabidopsis*, and other eight representative plants species were assessed at first. According to previous studies, *A. thalianan* contained 12 MTPs (AtMTP1-12). Compared with *Arabidopsis*, *N. tabacum* genome carried multiple MTP homologs for each AtMTP, but the homologs for AtMTP2 and AtMTP3 were absent. This result indicated that the *NtMTP* gene family might have undergone gene expansion and/or gene loss in the evolutionary history, probably due to the polyploidization events. In addition, *N. tabacum* was found to have the largest number of *MTP* family members among all plant species studied here, which is probably due to the large size of the *Nicotiana* genomes. There were eight, seven, and thirteen *NtMTP* genes belonging to Zn-CDFs, Zn/Fe-CDFs, and Mn-CDFs, respectively. Considering the implications of phylogenetic distributions in inferring structure and functional roles across species (Vatansever et al., 2017), these results would provide clues to uncover the functional characteristics especially the substrate-specificities of NtMTP proteins.

The characteristics of the *NtMTP* genes, including CDS length, protein size, MW, pI, GRAVY, sub-cellular localization and TMD number, were analyzed and predicted later. Consistently with previous study (Vatansever et al., 2017), NtMTP proteins were mainly predicted to be localized to vacuole, whereas some of them might also be localized in nucleus or cellular membrane, suggesting that NtMTPs might function as the vacuole-localized cation transporters. However, unlike other plant MTP families, of which the MTP12 had the biggest molecular size (Li et al., 2018), NtMTP6.1 and NtMTP6.2 were approximately four times the size of other NtMTPs, and two times the size of NtMTP12.1 and NtMTP12.2, respectively (Table 1). In addition, nearly half of the NtMTP proteins did not possess typical numbers of TMDs, especially for NtMTP 6.1 and NtMTP6.2. The obvious sequence differences between NtMTP6.1/6.2 and other NtMTPs indicated that these two proteins may have distinct biological functions and evolutionary processes which require further verification.

Besides the transmembrane region, the modified signature sequence between TMDs I and II (Paulsen and Saier, 1997; Montanini et al., 2007) and the characteristic C-terminal cation_efflux domain are two structural features of MTP proteins. Our results showed that all the NtMTP proteins contained these two typical structural characteristics. Moreover, the consensus residues HxxxD and DxxxD were also identified in corresponding members of three major substrate-specific groups, which were in accordance with and provided a valuable support for our phylogeny assays. Furthermore, ZT_dimer was reported to be the dimerization region of the whole molecule of zinc transporters, as the full-length members formed a homodimer during activity (Lu and Fu, 2007). The presence of ZT-dimer in certain NtMTPs suggested that these proteins might need to form homodimers or heterodimers when serving as metal ion transporters. In addition, SpoIIIAC, which was encoded by motif 9, belonged to StageIII sporulation protein AC/AD protein family. This family consists of several bacterial SpoIIIAC and SpoIIIAD sequences, but the exact function of this family is unknown. SpoIIIAD is an uncharacterized protein which is part of the spoIIIA operon that acts at sporulation stage III as part of a cascade of events leading to endospore formation (Mizuno et al., 1996). Our identification of this motif indicated a novel function of corresponding NtMTP proteins other than cation transporter which need to be explored by future studies. Taken together, these structure features of NtMTP proteins were consistent with the canonical structure characteristics of MTP transporters. Meanwhile, these results also showed a structural similarity of NtMTPs within the same group but a distinction between different groups, indicating the conserved but diverse functions of NtMTP family.

Previous studies suggested that functional redundancy may induce gene loss (Lynch and Conery, 2000; Qian et al., 2010). In the present study, the gene member size of *MTP8* was the largest compared with other *MTPs* in both *N. sylvestris* and *N. tomentosiformis* genome, which may lead to functional redundancy among *MTP8* paralogs in their progeny *N. tabacum* genome and induce gene loss after polyploidization. This may appear to explain well the inconsistency of the expected and actual gene number of group 8 MTPs in *N. tabacum*. On the other hand, gene duplication has been recognized as a major source of new genes, and has contributed to the evolution of novel functions (Hittinger and Carroll, 2007; Panchy et al., 2016). Apart from whole-genome duplication (WGD), gene duplication could be derived from subgenomic duplication events, such as tandem and segmental duplication (Bailey et al., 2002; Zhang, 2003). By using bioinformatics methods, *NtMTP7.1*/*NtMTP7.2* was found as segmental duplication event in this study, which might result in the expansion of group 7 *NtMTPs*. Nevertheless, it is worth noting that, due to the limited chromosome localization information of *NtMTP* genes, the number of gene duplication events would be underestimated.

The potential regulatory mechanisms controlling *NtMTPs* gene expression were explored both by analyzing the CREs and the microRNA target sites in the promoter regions and the coding sequences of *NtMTP* genes, respectively. Finally, a total of 2269 putative CREs involved in multiple biological processes and eight NtmiRNAs were identified. Previous studies showed that some of these identified miRNAs were involved in both abiotic and biotic stress response. For example, the expression of nta-miR172, nta-miR479, and nta-miR397 would be regulated by topping and wounding treatments (Guo et al., 2011; Tang et al., 2012). In addition, nta-miR6019 and nta-miR6020 were reported to guide cleavage of transcripts of the Toll and Interleukin-1 receptor-NB-LRR immune receptor *N* from tobacco that confers resistance to tobacco mosaic virus (TMV), and might also respond to Cd stress through negatively regulating their target genes (Li et al., 2012; He et al., 2016). Thus, it would be of great interest to explore the functions of *NtMTP* genes in these hysiological processes in future studies.

Tissue expression pattern analysis provided valuable clues about the important roles of *NtMTP* genes in tobacco growth and development. For instance, *NtMTP9.1* and *NtMTP9.2* were exclusively expressed in young flower, whereas *NtMTP8.5* was most abundant in all three types of leaf, indicating that they might play roles in early flower development and leaf development, respectively. Interestingly, although *NtMTP* genes within most groups showed similar tissue expression patterns, those of members from group 8 were somehow different. *NtMTP8.1* and *NtMTP8.2* were highly expressed in both young and mature flowers, indicating that they might be crucial for tobacco flower development. Besides, the expression levels of *NtMTP8.1* and *NtMTP8.4* were decreased during leaf maturation and senescence which suggested that these two genes might be involved in regulating tobacco leaf development. However, contrary to other *NtMTP8* genes, *NtMTP8.3* was not or rarely expressed in all tissues examined. Qian et al. proposed that expression reduction, as a special type of subfunctionalization, could facilitate the retention of duplicates and the conservation of their ancestral functions (Qian et al., 2010). Hence, the relative low gene expression of *NtMTP8.3* might be beneficial to retain its biological functions and avoid gene loss during evolution processes. The reliability of the transcriptome data was further validated by qRT-PCR. It is undeniable that there was some inconsistency between the transcriptome data and our qRT-PCR results. This may due to the different tobacco varieties and growth conditions used for sampling, which would likely affect the expression patterns of *NtMTPs*.

It is noteworthy that although the gene expression patterns in response to different stresses would suggest the functional roles of corresponding genes, the changes of *MTPs* gene transcripts responses to their potential metal substrates supply were diverse and complicated. AtMTP1, which encodes a tonoplast-localized Zn transporter, was found to be steady when exposed to excess Zn both at transcription and translation levels (Dräger et al., 2004; Kobae et al., 2004). Moreover, the expression of *CsMTP1* from cucumber was not affected by elevated Zn concentration, although the level of protein encoded by this gene was increased significantly in metal excess (Migocka et al., 2015a). As already mentioned, AtMTP12 could form a heterodimeric complex with AtMTP5 to transport Zn, however, the accumulation of *AtMTP12* does not depend on Zn concentration (Fujiwara et al., 2015). And similar results were also described in cucumber in a recent publication (Migocka et al., 2018b). Furthermore, the expression of all four genes from Mn-CDFs (*AtMTP8*, *AtMTP9*, *AtMTP10*, and *AtMTP11*) was little affected by Mn^2+^ supplies that ranged from basal to severely toxic (Delhaize et al., 2007). Similarly, in our study, apart from *NtMTP1.2* and *NtMTP4.1*, the gene expression levels of *NtMTPs* in Zn-CDFs were largely unchanged in the presence of excess Zn. And also in Zn/Fe-CDFs, only *NtMTP6.1* and *NtMTP6.2* were up-regulated by Zn and down-regulated by Fe in tobacco leaves, respectively. Hence, on the one hand, it would be necessary to investigate the responses of *NtMTPs* to metal ions at the protein levels. On the other hand, as the activity of both protein components of heterodimeric complexes is differentially regulated by Zn availability (Migocka et al., 2018b), the identification of the protein complexes in NtMTP family and the investigation of the regulatory mechanisms of the corresponding components under heavy metal supplies would be of great interest for future studies.

Yeast metal sensitivity test assay was a convient and commonly used method to determine the substrates of metal transporters. Our results showed that NtMTP1.2 was a Zn and Co trasporter, and NtMTP8.1, NtMTP8.4, and NtMTP11.1 functioned as Mn transporters in yeast cell. Moreover, NtMTP5.2 and NtMTP7.2 could not rescue the sensitivities of tested yeast mutants to corresponding metals. These results were consistent with those of previous studies (Shingu et al., 2005; Peiter et al., 2007; Fujiwara et al., 2015; Eroglu et al., 2016; Migocka et al., 2018b), except for NtMTP7.2. CsMTP7, which was the only functionally characterized MTP protein from Zn/Fe-CDFs to date, served as a highly specific mitochondrial Fe importer in both yeast and Arabidopsis protoplants (Migocka et al., 2018a). However, in the present study, NtMTP7.2 could not restore the growth of yeast mutant *ccc1*△ to excess Fe, indicating a function diversity of MTP7 protein among different plant species. In general, these results would provide important clues for clarifying the mechanism of heavy metal transport mediated by NtMTP proteins and the roles of NtMTPs in heavy metal tolerance and homeostasis.

## Conclusion

Twenty six, thirteen, and twelve *MTPs* in three main *Nicotiana* species (*N. tabacum*, *N. sylvestris*, and *N. tomentosiformis*) were identified, respectively, in the present study, and a comprehensive analysis of *NtMTP* genes was further carried out. The 26 NtMTPs were divided into three major substrate-specific groups (Zn-CDFs, Zn/Fe-CDFs, and Mn-CDFs) and seven primary groups (1, 5, 6, 7, 8, 9, and 12), and appeared to have underwent gene loss and expanded through segmental duplication after polyploidization. All the NtMTPs contained modified signature sequences and the cation_efflux domain, whereas some of them also harbored the ZT_dimer. The expression patterns of *NtMTP* genes in different tissues and in response to various heavy metal toxicity indicated the conserved and essential roles of *NtMTP* genes in tobacco growth and development, especially in heavy metal transport and tolerance. NtMTP8.1, NtMTP8.4, and NtMTP11.1 were found to function as Mn transporters in yeast cell. These results shed some light on the evolution of *MTPs* in tobacco as well as the regulatory mechanism controlling *NtMTPs* gene expression, and provided a valuable resource for better understanding the biological roles of *NtMTP* genes in tobacco.

## Author Contributions

JL and YfG conceived and designed the experiments. JL and YlG performed the experiments. JL analyzed the data. YT, DW, and XC contributed to reagents and equipments. JL wrote the manuscript. YfG and YY provided guidance on the whole manuscript. All authors reviewed and approved the final submission.

## Conflict of Interest Statement

The authors declare that the research was conducted in the absence of any commercial or financial relationships that could be construed as a potential conflict of interest.

## Footnotes

1 http://www.arabidopsis.org/

2 https://solgenomics.net/

3 http://www.ebi.ac.uk/interpro/

4 https://blast.ncbi.nlm.nih.gov/Blast.cgi

5 https://web.expasy.org/protparam/

6 http://www.csbio.sjtu.edu.cn/bioinf/plant-multi/

7 http://www.cbs.dtu.dk/services/TMHMM/

8 http://alternate.meme-suite.org/tools/meme

9 http://pfam.xfam.org/search#tabview=tab1

10 https://solgenomics.net/

11 https://github.com/CJ-Chen/TBtools

12 https://solgenomics.net/

13 http://bioinformatics.psb.ugent.be/webtools/plantcare/html/

14 http://plantgrn.noble.org/psRNATarget/

15 https://github.com/CJ-Chen/TBtools

16 http://www.euroscarf.de
