# Supplementary material for: Genome-Wide Identification, Comprehensive Gene Feature, Evolution, and Expression Analysis of Plant Metal Tolerance Proteins in Tobacco Under Heavy Metal Toxicity
Source: Front Genet. 2019 Apr 24;10:345. doi: 10.3389/fgene.2019.00345 (PMC6491887; doi:10.3389/fgene.2019.00345)
Supplement: Supplementary file 1 [file Table_1.docx]

**Table S1** Primer sequences of *NtMTP* genes used for qRT-PCR analysis

| Gene name | Forward primer (from 5’ to 3’) | Reverse primer (from 5’ to 3’) |
| --- | --- | --- |
| *NtMTP1.1* | GTTCAAGGCTTCCTCATCCGAC | GCTTCTTTTTCTTTTCACCTTCTGAT |
| *NtMTP1.2* | CATGGCGAGCATACGCATATAC | TCTTTTTCTTTTCACCTTCTGGC |
| *NtMTP4.1* | AAGAAGAATCAATTTTGGCGTG | CTTTATTTCAGACTTGGAATCCTCA |
| *NtMTP4.2* | CCATTACCAATTACCAATAAGAAGAAT | TTTATTTCAGACTTGGAATCCTCC |
| *NtMTP5.1* | ATCTGAGCACAAGCATTACTTGATT | CAGCATTCCAAGTGTCAACAGC |
| *NtMTP5.2* | TCTGAGCACAAGCATTACTTGATC | TAAGACTTCAGCATTCTTCACCC |
| *NtMTP6.1* | GATATCAGAGATCTGAGATATGTTTGAA | CAACAGTTGGTGGAATGTGTATAGT |
| *NtMTP6.2* | TGGTTGATGCTGCTATTCCTTC | CGTCAACCCATGTTTCTCAAAT |
| *NtMTP7.1* | GGAATGGTATGTGATGCTAAACCT | CATAGATTGACATCAGTTTCAGTAATG |
| *NtMTP7.2* | ATGGACAGGGTTCTGCAGTTC | CATAGATTGACATCAGTTTCAGTAATT |
| *NtMTP7.3* | TCCATATGGTTACTTTAGGAAACGT | TTGTATTGCCACTATAAGCGAAAC |
| *NtMTP8.1* | GGCTGTTTCGATGCTGGAGTT | TAACTGAGTTTCGACGCGCAT |
| *NtMTP8.2* | GAAGTTCCAAATGTCATACCAAA | GATCTTAAAGGCAAGTAATAAGACA |
| *NtMTP8.3* | GAGGGAGAATTAGAGGCTAACAATA | CACCTTATCTGGAAGCCTAGACAA |
| *NtMTP8.4* | GTCTTGTGTTAATGATAATGGAGAAAA | ATAGGTGAGTCAACCACAGTATGG |
| *NtMTP8.5* | TGGTGATGTTGAATCTTGCTC | GATATTTTTCATTGATAGGTGAGTCAG |
| *NtMTP9.1* | TGAATCTGGCTTTTTACCTGGA | ACCGATGCAAAAACAATAATACC |
| *NtMTP9.2* | TCAATGAGATGGACACTGATGAA | CCGTATTAGGACGAGACACAGG |
| *NtMTP10.1* | GTAATTCCTCAGCTCATGCCAT | CCATATTTGCTATGTTTGACACAA |
| *NtMTP10.2* | CAACATGCTAAGAGCGAACGA | TGACCACAGTGACAGAAACCATT |
| *NtMTP10.3* | TTCGAGCATATACTTTTGGTGCT | AAAAGCTCGCTCAACTTCAGG |
| *NtMTP10.4* | TAAGTCTCGCCCTGAGATGGAT | GCCGTCACTAATCCAACTGAGTTA |
| *NtMTP11.1* | TCATCTTGACTATGAATACAGCCAT | TTTTAAAGAGCAATCACCAGATTAG |
| *NtMTP11.2* | TCATCTTGACTACGAATACAGCCAC | TTAAGGAGCAATCACCAGATCAA |
| *NtMTP12.1* | AATCAAGTACAAGGGATGGCTT | GAGTTAGTGAAGCTCCAAAAATGA |
| *NtMTP12.2* | AATCAAGTACAAGGGATGGCTC | TTCGTGAAGCTCCAAACATGC |
| *NtL25* | CAAAAGTTACATTCCACCGACC | TGTCCACAATGAAAACAAGGGT |
| *Ntubc2* | TTTTAAGCGGTTACAGCAGGAT | CACAGTTGGTGGTTTGTTTGG |
