# Supplementary material for: Genome-Wide Identification, Comprehensive Gene Feature, Evolution, and Expression Analysis of Plant Metal Tolerance Proteins in Tobacco Under Heavy Metal Toxicity
Source: Front Genet. 2019 Apr 24;10:345. doi: 10.3389/fgene.2019.00345 (PMC6491887; doi:10.3389/fgene.2019.00345)
Supplement: Supplementary file 2 [file Table_2.docx]

**Table S2** Primer sequences of *NtMTP* genes used for yeast expression plasmids construction

| Primer name | Primer sequence (from 5’ to 3’) | Restriction enzyme site |
| --- | --- | --- |
| NtMTP1.2 pYES2-F | CGGGGTACCATGGAGACGCAGAACCTGG | *Kpn*I |
| NtMTP1.2 pYES2-R | GCTCTAGATTACTCTCTTTCTATTTGAATGG | *Xba*I |
| NtMTP5.2 pYES2-F | CGGGGTACCATGAATCAGAGTCATACGCC | *Kpn*I |
| NtMTP5.2 pYES2-R | CCGGAATTCTCATGTATAATCAATTTGTACGG | *Eco*RI |
| NtMTP7.2 pYES2-F | CGGGGTACCATGCCTAACCCTCGTGTTCT | *Kpn*I |
| NtMTP7.2 pYES2-R | CCGGAATTCTCATGGGGATGGTCCAATTG | *Eco*RI |
| NtMTP8.1 pYES2-F | CGGGGTACCATGGATGTGGAAGAGCCATTG | *Kpn*I |
| NtMTP8.1 pYES2-R | GCTCTAGATTAAGGTGGGCTGTTGGGAA | *Xba*I |
| NtMTP8.4 pYES2-F | CGGGGTACCATGGAGGGAGAAGAAGTAAAGAATG | *Kpn*I |
| NtMTP8.4 pYES2-R | GCTCTAGACTAAGGTTCAGTGTTTGGTAGCC | *Xba*I |
| NtMTP11.1 pYES2-F | CGGGGTACCATGTTGGAAGTAGTACCTCTTCA | *Kpn*I |
| NtMTP11.1 pYES2-R | CCGGAATTCCTATTGGTATGCCTGTGCAT | *Eco*RI |

Notes: The sequence of restriction enzyme site in the primer was underlined.
