# Supplementary material for: Genome-Wide Identification, Comprehensive Gene Feature, Evolution, and Expression Analysis of Plant Metal Tolerance Proteins in Tobacco Under Heavy Metal Toxicity
Source: Front Genet. 2019 Apr 24;10:345. doi: 10.3389/fgene.2019.00345 (PMC6491887; doi:10.3389/fgene.2019.00345)
Supplement: Supplementary file 4 [file Table_4.docx]

**Table S4** The sequences and the Pfam annotations of conserved motifs in NtMTP proteins

| Motif ID | Motif sequence | Length | NSites | Evalue | Pfam |
| --- | --- | --- | --- | --- | --- |
| MEME-1 | LMJYCRRFKNEIVRAYAQDHFFDVITNSVGLVAAVLAIRFYWWIDPVGAIIJALYTISTWAKTVJENVWSLIGRSAPPEFLQKLTYLI | 88 | 12 | 0 | Cation_efflux；PF01545, Cation efflux family |
| MEME-2 | GFILWFTHLSMKNPNQYKYPIGKKRMQPV | 29 | 13 | 6.50E-260 | No motif was found in Pfam. |
| MEME-3 | DTVRAYTFGVHYFVEVDIVLPEDMPLKEAHNIGETLQEKLEQLPEVERAFVHLDFEC | 57 | 10 | 0 | ZT_dimer；PF16916, Dimerisation domain of Zinc Transporter |
| MEME-4 | LFAAKIYASVKSGSLAIIASTLDSLLDLL | 29 | 21 | 7.80E-224 | Cation_efflux；PF01545, Cation efflux family |
| MEME-5 | RKQGKIAEYYKKQERLLEGFNEMDTINESGFLPGSLTED | 39 | 8 | 4.90E-138 | No motif was found in Pfam. |
| MEME-6 | MTSEKEKWLIGIMVSVTVVKL | 21 | 13 | 1.50E-114 | No motif was found in Pfam. |
| MEME-7 | GIIIFAAVMATLGFQVLVZAVEZLIENSR | 29 | 8 | 5.20E-83 | No motif was found in Pfam. |
| MEME-8 | SRWPPBGEHPYGYGRFETLGA | 21 | 16 | 6.10E-66 | Cation_efflux；PF01545, Cation efflux family |
| MEME-9 | DLLCTIFFSVLVLSTVIPLLRSILEILLZRTPREIDATRLEK | 42 | 8 | 4.50E-65 | SpoIIIAC；PF06686, Stage III sporulation protein AC/AD protein family |
| MEME-10 | EKKKKHTNINIEGAYLHVJADTIQSVGVMIAGAJIWYKP | 39 | 6 | 4.80E-68 | Cation_efflux；PF01545, Cation efflux family |
