# Supplementary material for: Genome-Wide Identification, Comprehensive Gene Feature, Evolution, and Expression Analysis of Plant Metal Tolerance Proteins in Tobacco Under Heavy Metal Toxicity
Source: Front Genet. 2019 Apr 24;10:345. doi: 10.3389/fgene.2019.00345 (PMC6491887; doi:10.3389/fgene.2019.00345)
Supplement: Supplementary file 5 [file Table_5.docx]

**Table S5** Gene name and ID of *MTP* in *N. sylvestris* and *N. tomentosiformis*

| *N. sylvestris* | |  | *N. tomentosiformis* | |
| --- | --- | --- | --- | --- |
| Gene name | Gene ID |  | Gene name | Gene ID |
| *NsylMTP3* | mRNA_33190_cds |  | *NtomMTP4.1* | >mRNA_22564_cds |
| *NsylMTP4* | mRNA_38409_cds |  | *NtomMTP5.1* | >mRNA_41661_cds |
| *NsylMTP5* | mRNA_65833_cds |  | *NtomMTP6.1* | >mRNA_84742_cds |
| *NsylMTP6* | mRNA_16032_cds |  | *NtomMTP7* | >mRNA_20210_cds |
| *NsylMTP7* | mRNA_64216_cds |  | *NtomMTP8.1* | >mRNA_45203_cds |
| *NsylMTP8.1* | mRNA_58740_cds |  | *NtomMTP8.2* | >mRNA_32327_cds |
| *NsylMTP8.2* | mRNA_73424_cds |  | *NtomMTP8.3* | >mRNA_7623_cds |
| *NsylMTP8.3* | mRNA_77477_cds |  | *NtomMTP9* | >mRNA_28204_cds |
| *NsylMTP9* | mRNA_73530_cds |  | *NtomMTP10.1* | >mRNA_74724_cds |
| *NsylMTP10.1* | mRNA_59783_cds |  | *NtomMTP10.2* | >mRNA_69177_cds |
| *NsylMTP10.2* | mRNA_62394_cds |  | *NtomMTP11* | >mRNA_59462_cds |
| *NsylMTP11* | mRNA_33648_cds |  | *NtomMTP12* | >mRNA_62570_cds |
| *NsylMTP12* | mRNA_6981_cds |  |  |  |
