# Supplementary material for: Genome-Wide Identification, Comprehensive Gene Feature, Evolution, and Expression Analysis of Plant Metal Tolerance Proteins in Tobacco Under Heavy Metal Toxicity
Source: Front Genet. 2019 Apr 24;10:345. doi: 10.3389/fgene.2019.00345 (PMC6491887; doi:10.3389/fgene.2019.00345)
Supplement: Supplementary file 7 [file Data_Sheet_1.docx]

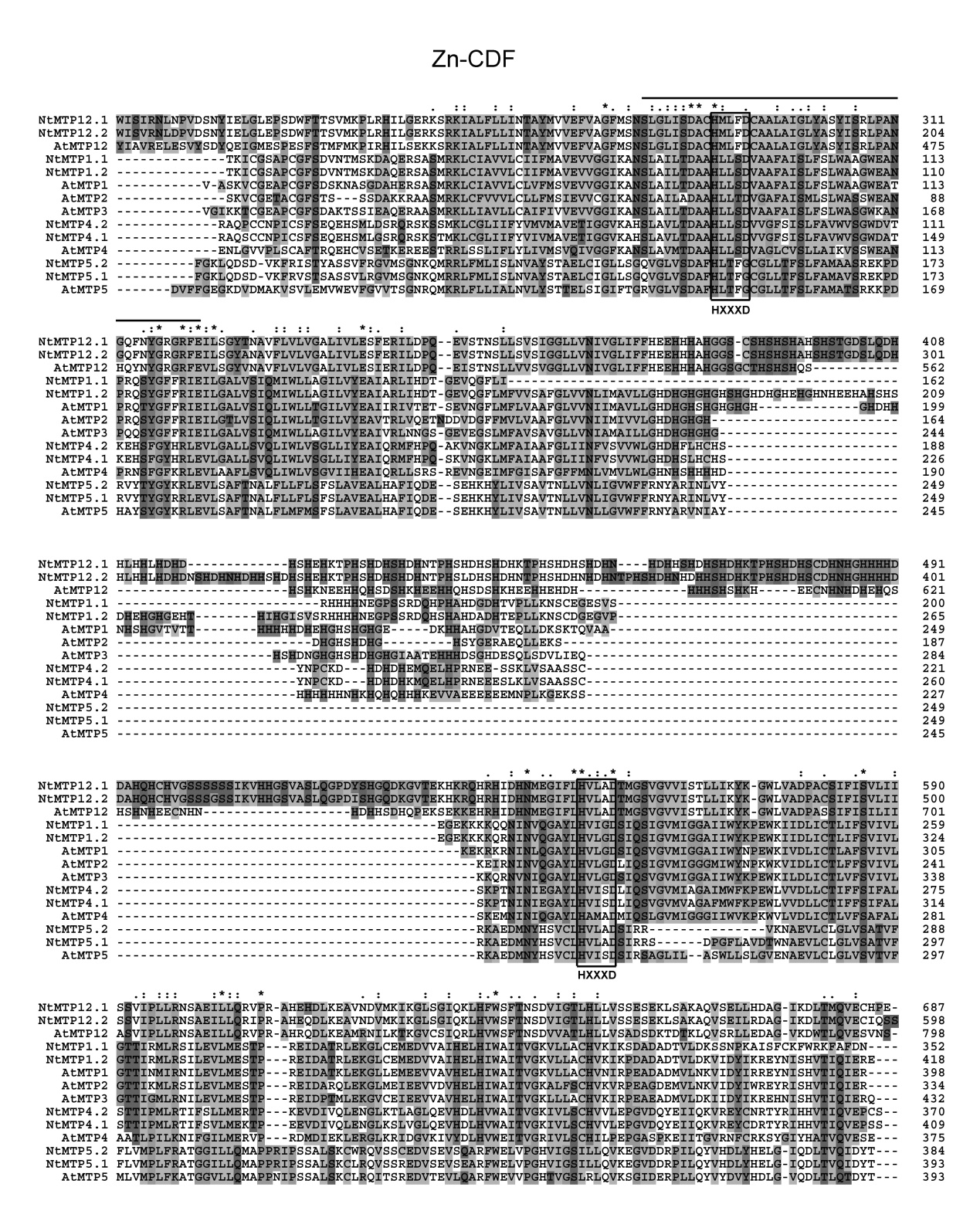


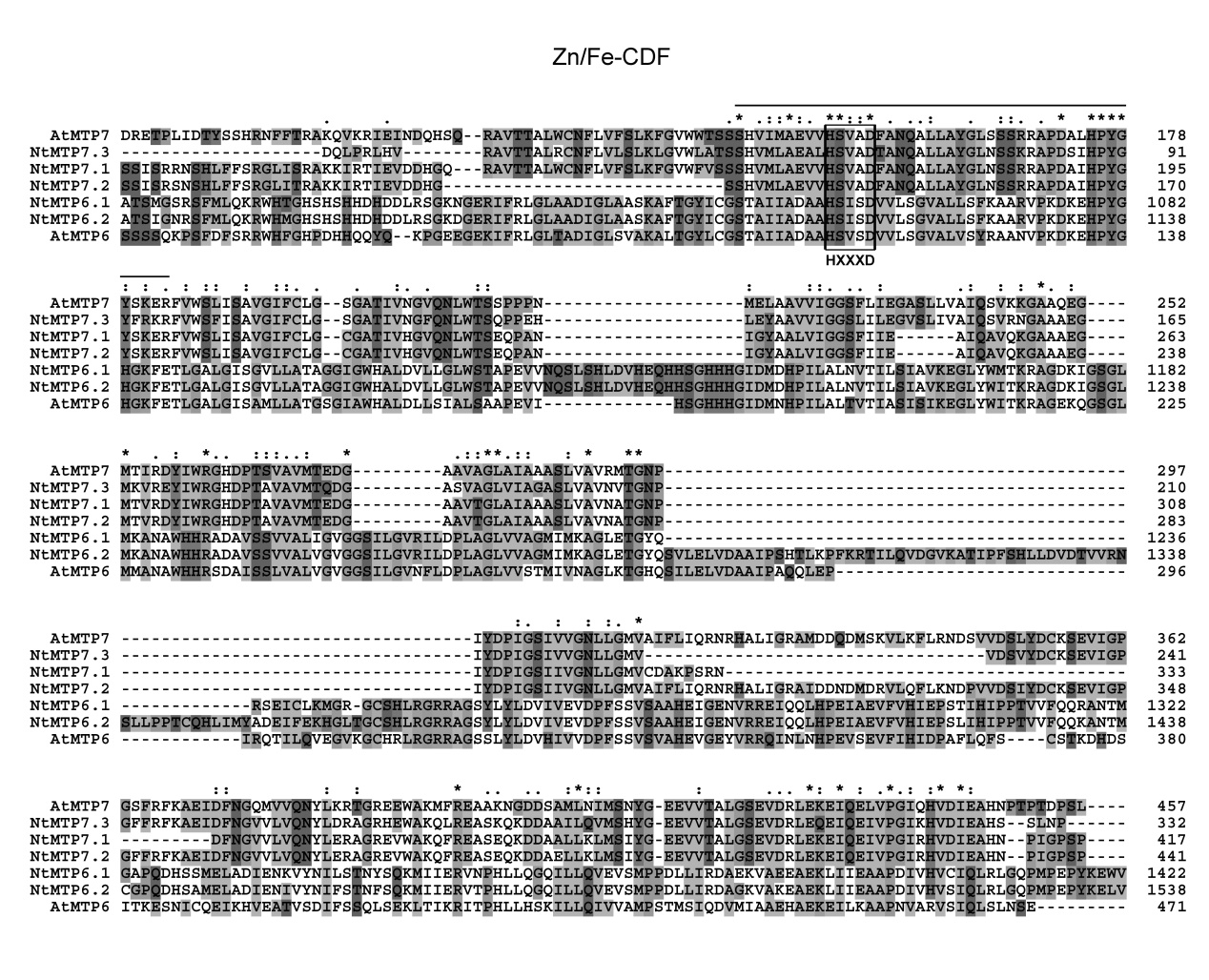


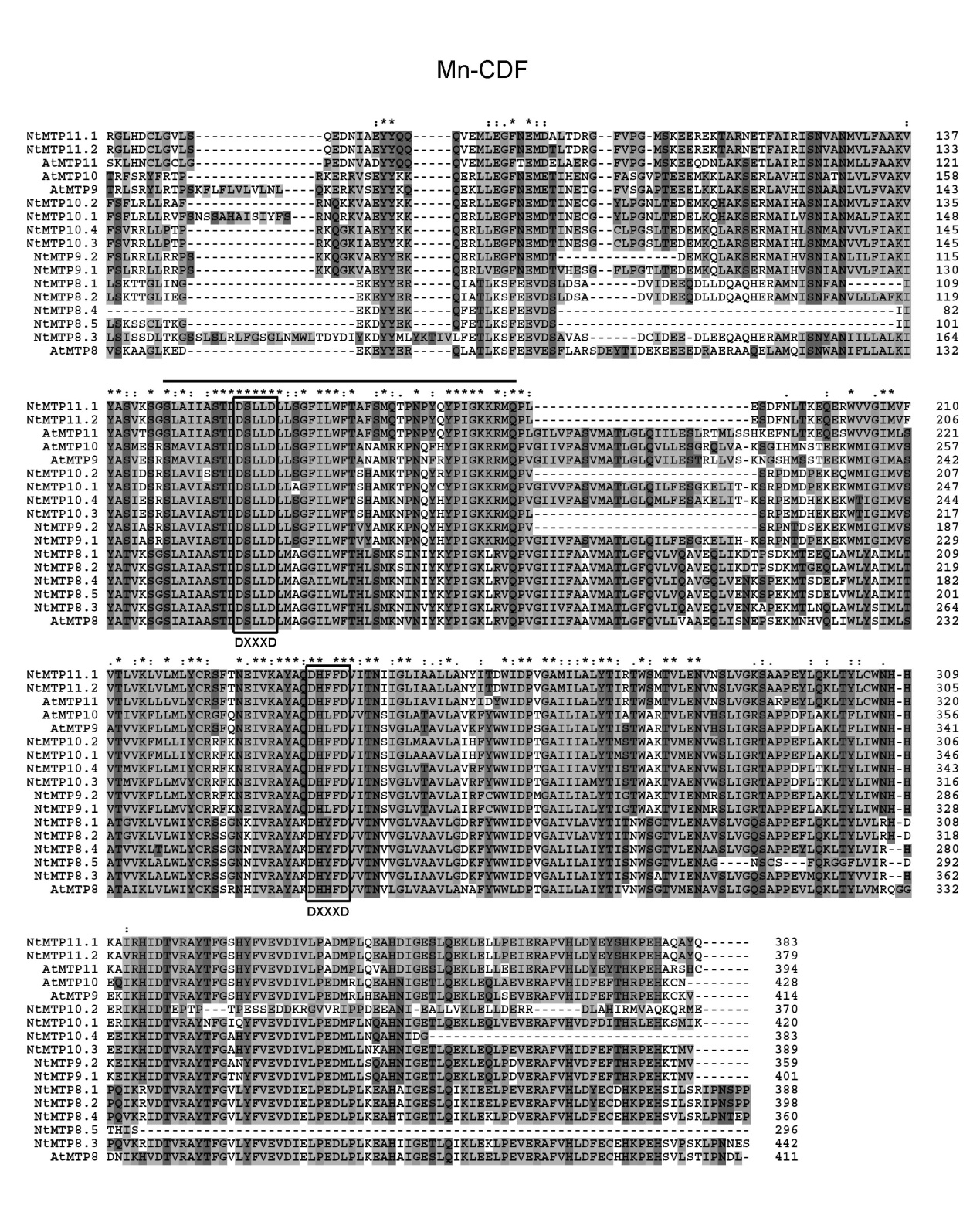


**Figure S1** Multiple sequence alignment of NtMTP and AtMTP proteins.

‘*’ indicates positions which have a single, fully conserved residue. ‘:’ indicates that one of the following 'strong' groups is fully conserved: STA, NEQK, NHQK, NDEQ, QHRK, MILV, MILF, HY, FYW. ‘.’ indicates that one of the following 'weaker' groups is fully conserved: CSA, ATV, SAG, STNK, STPA, SGND, SNDEQK, NDEQHK, NEQHRK, FVLIM, HEY. The signature sequences and the consensus sequence HXXXD or DXXXD (X=any amino acid) are indicated with black line and open boxes, respectively.
